# Comparative study of abdominal and thoracic aortic aneurysms: their pathogenesis and a gingival fibroblasts-based ex vivo treatment

**DOI:** 10.1186/s40064-015-0976-9

**Published:** 2015-05-16

**Authors:** Hafida Cherifi, Bruno Gogly, Ludwig-Stanislas Loison-Robert, Ludovic Couty, François Côme Ferré, Ali Nassif, Antoine Lafont, Benjamin PJ Fournier

**Affiliations:** Centre de recherche des cordeliers, INSERM UMRS 1138, Team 5, Laboratory of Molecular Oral Pathophysiology, Paris, France; Paris centre de recherche cardiovasculaire, INSERM UMRS 970, Team 11, Paris, France; Paris-Est University, Créteil, France; Paris-Descartes University, Paris, France; Paris-Diderot University, Paris, France; A. Chenevier/H.Mondor Hospitals, Dental Department, APHP, Créteil, France; Rothschild Hospital, Dental Department, AP-HP, Paris, France

**Keywords:** Gingival fibroblast, Aortic aneurysm, Cell therapy, Porphyromonas gingivalis

## Abstract

Aortic aneurysms (AAs) consist of slow proteolysis and loss of both collagen and elastin matrix in the aorta wall, leading to wall dilation, weakening and rupture in well-advanced lesions. This can occur in both abdominal aorta (Abdominal Aortic Aneurysm: AAA) and thoracic aorta (Thoracic Aortic Aneurysm: TAA). To date, no non-surgical therapy has been proposed to slow or stop AA progression. Previously published preclinical studies from our team using an aneurysm rabbit model showed a promising concept for treatment of AAs with gingival fibroblast (GFs) which are readily available cells. In this study, we investigated the possible tissue repair of human AAAs and TAAs using *ex vivo* models co-cultured with GFs. Histological analysis showed that TAA and AAA are two distinct pathologies. Both lesions presented destruction of the aorta wall, highly evidenced in AAA samples. The results have confirmed the presence of the bacterial Porphyromonas *gingivalis* (Pg) protein in all AAA samples, but not in TAA samples, indicating the possible role of an infectious factor in the developing and progression of AAA lesions compared to TAA. The co-culture of GFs with AA lesions shows increased expression of TIMP-1, the inhibitor of the aneurysm severity marker MMP-9. Our study indicates that GFs might ameliorate aorta wall reestablishment in both AA types by their regenerative and immunomodulatory capacities. It also demonstrates the possible infectious cause of AAA compared with TAA that may explain their different behavior.

**Electronic supplementary material:**

The online version of this article (doi:10.1186/s40064-015-0976-9) contains supplementary material, which is available to authorized users.

## Introduction

Human gingiva has a unique ability to heal. This potential for regeneration may be explained by the presence of sub-populations of stem cells recently isolated and expanded from this tissue (Fournier et al. 2010; Egusa et al. 2010; Tomar et al. 2010). Currently, human gingival fibroblasts (GFs) are successfully implicated for cell therapy of many organs including cornea, trachea, urethra, vocal cords and in the reconstruction of eyelids (Fournier et al. 2013). Our team focuses on the use of gingival fibroblasts in cell therapy in the treatment of aortic aneurysms. This disease, becoming more common with the aging population, often does not receive real treatment because only a particularly invasive surgery procedure is offered in advanced cases of the disease (Sakalihasan et al. 2005). Aneurysms are degenerative pathologies caused by the destruction of the extracellular matrix, including elastic fibers (Shah 1997; Sakalihasan et al. 1993). This leads, in well-developped cases, to a rupture of the aortic wall (Dobrin & Mrkvicka 1994). Proteolytic enzymes, including matrix (Petersen et al. 2000; Barbour et al. 2007). The elastase MMP-9 is particularly studied and appears elsewhere as a marker of the severity of this pathology. Our previous studies have clarified the roles of MMP-1, MMP-3 (Naveau et al. 2007), MMP-7 (Gogly et al. 2009), and MMP-9 (Gogly et al. 2009) in aortic aneurysm pathophysiology *in vitro* and *ex vivo* models. The role of MMP-9 has been particularly studied by our team and the development of an animal model of aneurysms in rabbits helped to understand the molecular mechanisms involved in the pathology and therefore consider therapeutic strategies (Durand et al. 2012). Our studies of GF-based cell therapy showed primary evidence of the therapeutic potential of TIMP1; over-expressed by GFs in our different models (Naveau et al. 2007; Gogly et al. 2009; Durand et al. 2012). However, all of these studies are not fully representative of the complex pathophysiology of human aneurysmal disease. In humans, AAs may be distingued based on their location: aneurysms of the abdominal aorta (AAA) and aneurysms of the thoracic aortic (TAA). Both present distinctive pathological and histological profiles (Guo et al. 2006). Their etiopathology is multifactorial and is still not well-defined. Atherosclerosis is considered the most common cause of AAs (Sakalihasan et al. 2005; Guo et al. 2006), even though several reports have strongly suggested that AAs may be caused by genetic factors, more precisely for TAA (Hoel 2013). The relationship between the pathogenic bacteria that cause atherosclerosis, including those present in oral cavity and causing periodontal diseases, and the subsequent development of AA has been well established (Cook & Lip 1996; Kurihara et al. 2004). We hope, in this new research, to complete our previous findings by studying the main characteristics of AAA and TAA and the presence of oral bacteria (PG, TD, TF, PI) which could correlate the pathophysiology of the samples. We then reproduced our experiments published on co-cultures of gingival fibroblasts but in presence of human samples of AAA and TAA (rather than animal models). We observed particular TIMP-1 overexpression and inhibition of MMP-9.

## Material and methods

### Collection and culture of AA specimens

Samples of full-thickness aortic wall were collected from surgical wastes of 17 patients undergoing AAA (n = 11) and TAA (n = 6) surgery (Additional file 1: Table S1). Tissue collections were obtained with patient approval at Georges Pompidou European Hospital, Paris. Tissues were recovered in Dulbecco’s Modified Eagle Medium (DMEM) with 10 % Fetal Bovine Serum (FBS). Each fragment was divided into equal aorta wall samples of 4 mm diameter and embedded in 10 mL of 2 mg/mL collagen mixture and then cultured. This culture was previously described (Naveau et al. 2007; Gogly et al. 2007). The supernatant was analysed at day 1, 7 and 14 of culture. 24 hours before retrieving the supernatant, 10 % FBS DMEM was replaced by 0 % FBS DMEM to avoid serum interference.

### Histological analysis

Histological analyses were done using paraffin embedded sections of AAA (n = 5) and TAA (n = 7). The sections were collected from the archives of the pathological department of Georges Pompidou European Hospital and were a kind gift of Pr Bruneval. Orcein and Sirius red stainings were respectively used to localise the elastic fibers and collagen fibers.

SMC density analysis was done by immunofluorescence using primary antibody anti-alpha smooth muscle actin (ASMA) at 1:50 dilution (Dako®) and anti-mouse Alexafluor 594 secondary antibody F(ab)2 (red) (Abcam®) at 1:500 dilution.

Intensity of elastic and collagen fibers as well as cell countings were performed using Image J® software as previously described (Durand et al. 2012).

### Evaluation of bacterial presence in aneurysm

#### PCR

RNA from the paraffin embedded aneurysm samples were isolated using the FFPE RNeasy kit, (Qiagen®), and reverse transcribed using SuperScript II (Invitrogen®). Real time PCR was conducted using SYBER Green kit with specific primers (see Additional file 1: Table S2 for sequences (Kuboniwa et al 2004; Suzuki et al 2004)).

Dental plaque collected from one patient with active periodontitis was used as positive control, whereas samples collected from periodontal healthy patients (n = 2) served as negative control. RNA extraction of the dental plaque samples was realized using RNeasy kits (Qiagen®).

#### Immunohistochemistry (IHC) and Immunofluorescence

Bacterial Pg primary monoclonal antibody was recovered from 61BG1.3 mouse strain obtained from the Developmental Studies Hybridoma Bank developed under the auspices of the NICHD and maintained by The University of Iowa, Department of Biology, Iowa City, IA 52242. Briefly, following deparaffinization and rehydration of the tissue sections, samples for IHC were incubated with primary Pg antibody at 1:50 dilution for 1.5 h at 37 °C using Universal Quick Kit Vectastain (Vector®). IHC sections were then incubated with anti-mouse IgG secondary antibody for 10 minutes at 1:500 dilution. Stain development was achieved using Vector, NovaRED substrate kit. Sections for Immunofluorescence were incubated with fluorine ALEXA 594 (red) secondary antibody for 30 minutes at 1:500 dilution. Nuclear assessment was done by DAPI staining.

### Collection of GF and coculture of GF and AA specimens

Clinically healthy gingival samples were collected from surgery wastes from patients attending the dental department of Albert Chenevier hospital (Créteil, France); samples were cultured as previously described (Naveau et al. 2007) and the obtained GF were used in the following experiments.

The AA samples of 4 mm diameter were embedded in 10 mL of 2 mg/mL collagen mixture and then cultured with or without allogeneic GF. The supernatant was analysed at 7 and 14 days of coculture. 24 hours before retrieving the supernatant, 10 % FBS DMEM was replaced by 0 % FBS DMEM to avoid serum interference.

### TIMP-1, MMP-9/TIMP-1, MMP-9 secretion assays

MMP-9 secretion was analysed by zymography using a separation gel containing 0.2 % gelatin as previously described (Naveau et al. 2007; Gogly et al. 2007). The quantification was made by scanning the densitometry (Image J software) and expressed with density unit as previously described (Leber & Balkwill 1997). All MMP-9 isoforms could be detected (Pro-MMP-9, MMP-9/MMP-9, and active MMP-9). Elisa was used to specifically detect the concentration of active MMP-9, TIMP-1 and MMP-9/TIMP-1 complex in the supernatant thanks using their specific antibodies (R&D system).

### Statistical analysis

The data is presented as mean ± standard error of mean. Statistical analysis comparing two samples was conducted using the Mann-Whitney test. Statistical significance was set at p < 0.05.

## Results

### AAA and TAA are histopathologically different

**Fig. 1:**
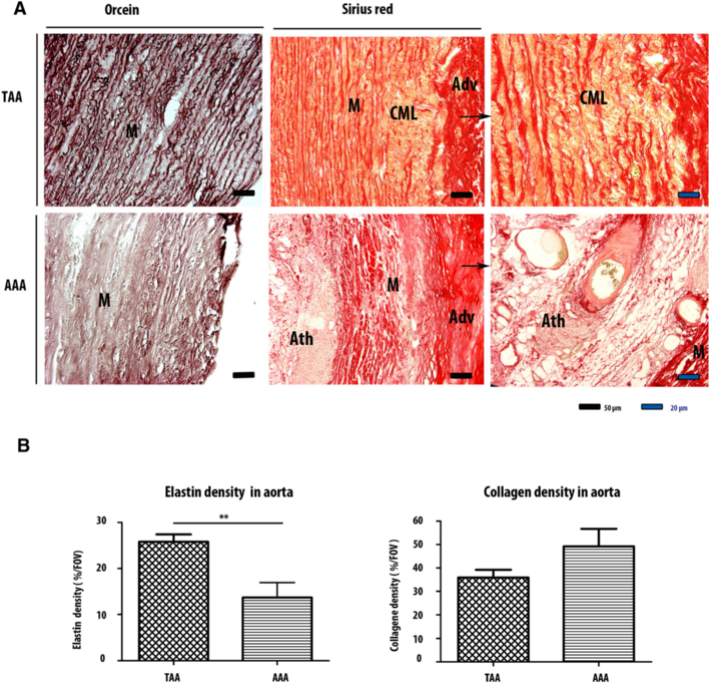
Elastic and collagen networks distribution in AAA and TAA. (**a**) Orcein staining shows a more important degradation of elastic network in AAA than in TAA. Sirius red staining indicates a fibrosis in AAA adventitia and also near atheroma. This staining also highlights the maintaining of the collagen network in TAA with small degradation areas known as cystic medial lesions (CML). (**b**) Elastic network is more degraded in AAA than in TAA (p = 0.003). There is not a significant difference between the collagen density of the both lesions. The difference is mainly qualitative. *p<0.1. **p<0.001. ***p<0.0001

We first noticed that the elastic fiber network of TAA was more conserved compared to AAA as highlighted by orcein staining (Fig. 1A). The TAA elastin field of view (FOV) was 25.82 % ± 1.593, and it was 13.68 % ± 3.261 for AAA (Fig. 1B). Collagen network was quite preserved in TAA with small degradation areas (Fig. 1A), while AAA collagen network was almost completely degraded. Fibrosis in adventitia and intima layers near the atheroma plaque was remarkable (Fig. A). When we compared the collagen FOV between AAA and TAA tunica media**,** no significant difference was found. The difference was mainly in adventitia.

**Fig. 2:**
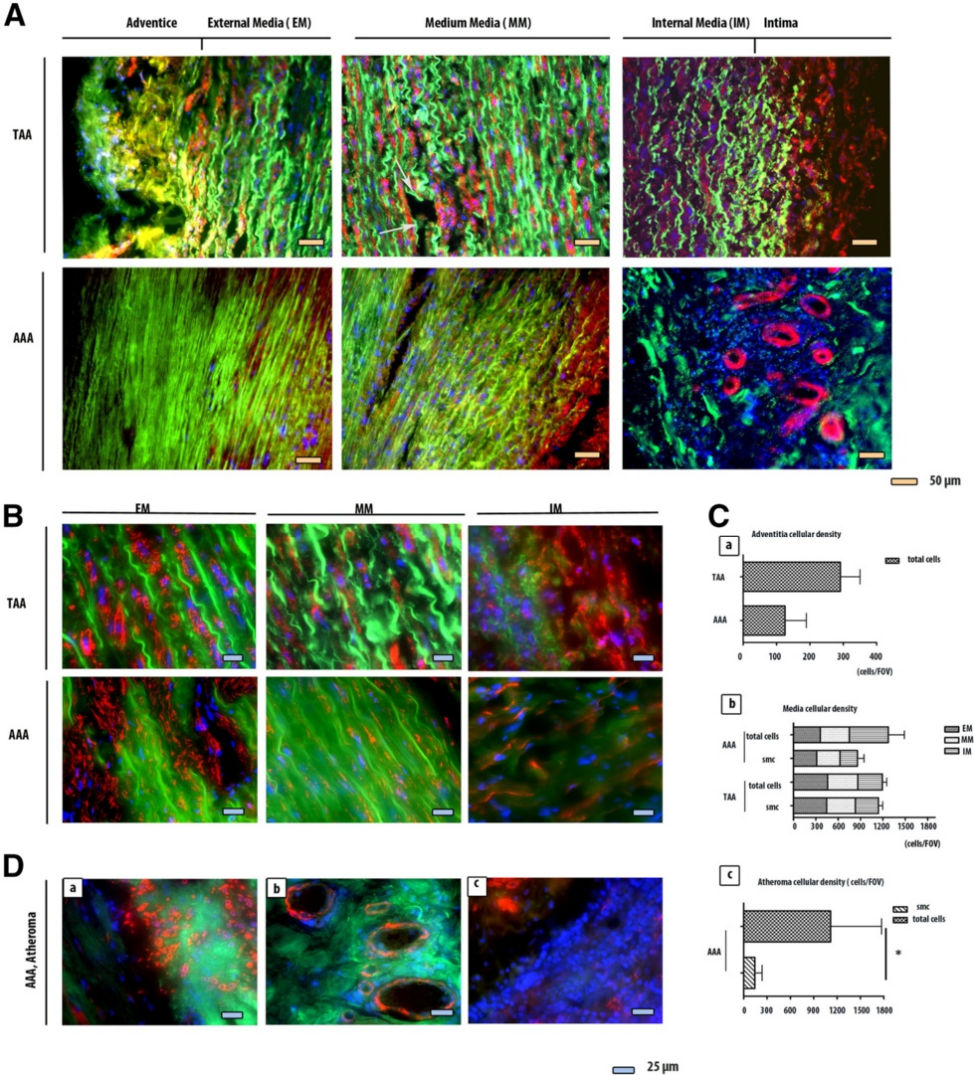
Comparaison between AAA and TAA lesions using α-smooth muscle actin (ASMA) immunofluorescence staining. (**a**) (**b**) The AAA’s external media, medium media and internal media are more degraded than TAA’s, especially the elastic fibers which auto fluoresce in green. For AAA, the elastic fibers do not have the curvilinear form that they have physiologically. For TAA, the network is interrupted by small afibrillar and acellular areas (white arrows). (**c**) SMC are stained in red (ASMA) with a blue nuclear (DAPI). They are present in both aneurysms. Their numbers is not significantly different between AAA’s and TAA’s medias or between the three areas of the media (Cb). The total cells number (DAPI+) is not that different between AAA’s and TAA’s medias (Cb). There are more cells in TAA’s adventitia than in AAA’s (p = 0.0498) (Ca). (**d**) For AAA, atheroma substitutes intima. The SMC are particularly confluent in some areas between internal media and atheroma (Da). They also organize themselves to turn into blood vessels (Db). Atheroma contains cell infiltration (Dc). Their number is more important than the SMC’s (p = 0.0092). *p < 0.1. **p < 0.001. ***p < 0.0001

Immunofluorescence results confirmed larger tissue degradation in the totality of the AAA wall compared to the TAA wall including adventitia, media and intima (Fig. 2A). The smooth muscle cells (SMC) in red and the elastic fibers in green, highlighted these differences**.** For TAA, discontinuities with acellular zones were found in the media. For AAA, the media was more affected, with destruction of the elastic network. The AAA adventitia was also affected with severe cellular loss (125.3cells/FOV ± 64.17) compared to TAA adventice (291.4 cells/FOV ± 59.03) (Fig. 2Ca). The TAA intima is not affected by the destruction compared to the AAA intima which is covered by atheroma and exhibits cell infiltration and neovascularisation (Fig. 2A).

We also studied the structure of the different levels of media: the external media (EM) (near adventice), the internal media (IM) (near intima) and the medium media (MM) (between both) (Fig. 2B). The area which seem most affected in the AAA compared to the TAA is the medium media and the internal media (Fig. 2B).

The cell density analysis suggests that there is no significant difference in total cell density and in smooth muscle cell density between TAA and AAA media layers (Fig. 2Cb). The variation of the cellular distribution between EM, MM and IM is not important in AAA or in TAA (Fig. 2Cb).

For intima, which is replaced by atheroma for AAA and not for TAA, the SMC number (1118 cells/FOV ± 654.7) is limited by the total number of cells (144.0 cells/FOV ± 92.36) (Fig. 2Cc). At higher magnification for the abdominal injury, we have detected particularly concentrated SMC groups between the internal media and atheroma (Fig. 2Da) with neovascularisation (Fig. 2Db) and cellular infiltration (Fig. 2Dc).

These results show that the severity of AAA tissue destruction is higher than TAA. We have researched what might be an aggravated factor for AAA. We suspected an infectious factor because contamination occurred in AAA sample cultures while it never occurred for TAA in which conditions were the same for all the samples. Our hypothesis was based on several reports demonstrating a possible association of periodontal infections with cardiovascular diseases (CVD) by elevated antibody titre to periodontopathic bacteria in CVD patients compared to non-diseased controls (Yamazaki et al. 2007; Chen et al. 2008; Suzuki et al. 2010).

Periodontitis is a bacterial-induced inflammatory disease where teeth progressively lose their supporting tissues. Several population-based studies have shown increased incidence of periodontitis with age starting from 35 years-old with high potential over the age of 65. We hypothesized that the presence of periodontopathic bacteria may affect AAA. We assumed that the infectious factor may increase the local inflammation and consequently the tissue degradation and also the healing process in our model. Therefore we have analysed thoroughly four main periodontal pathogens (Porphyromonas gingivalis (Pg), Treponema denticola (Td), Tannerella forsythia (Tf) and Prevotella intermedia (Pi) belonging to the red and orange complexes (Socransky et al. 1998).

As periodontitis and aorta aneurysms share MMP-9 as a pathological enzyme and as a biomarker, we wanted to know if the same bacteria could be involved in both diseases. Of course, we checked that our gingival fibroblasts cultures were devoid of such infectious factors (data not shown).

### Periodontopathic bacteria aggravate the severity of aorta aneurysm

**Fig. 3:**
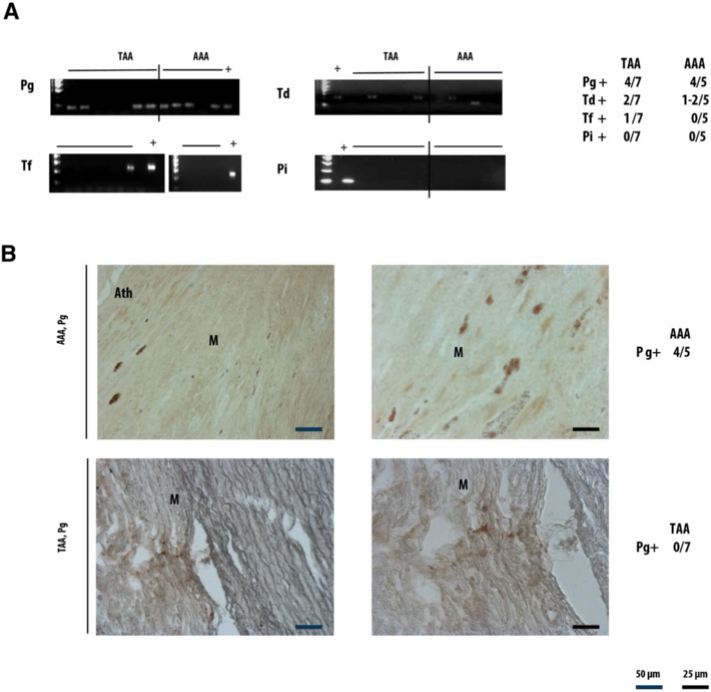
Presence of periodontopathic bacteria in AAA and TAA. (**a**) By RT- PCR, Pg and Td are detected in both aneurysms lesions and Tf only in TAA. Pi is not detected in any samples. Pg is the most frequent bacteria spotted in the samples. (**b**) Using Immunohistochemistry, Pg protein is detected in AAA but not in TAA

We studied by PCR the presence of periodontopathic bacteria, wondering if it could possibly migrate to the lesion areas of AAs (AAA n = 5 and TAA n = 7) and participate in aggravating the local inflammation or its maintenance. The results have shown that these pathogens are present in both types of aneurysms (Fig. 3A, Additional file 1: Table S3). Pg was detected in most of the studied samples. Tf bacterium was found only in one TAA sample, Td in two TAA samples and in two AAA samples. Pi was not observed in any studied samples.

Since Pg was detected in most samples and its presence was strongly confirmed by multiple PCR, we further investigated Pg by immunohistochemistry using a specific Pg antibody (Fig. 3B). This pathogen was clearly found in 4/5 AAA samples. However, we could not confirm its presence in any of the 7 TAA samples using this technique.

**Fig. 4:**
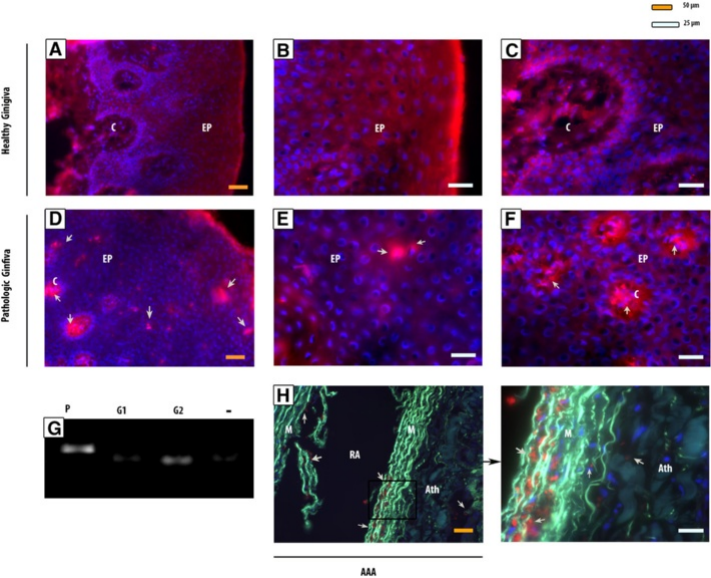
Pg detection in healthy and pathological gingiva, in dental plaque and in AAA. Pg bacteria (red Immunofluorescence staining) is not spotted in healthy gingiva (**a**), nor in the epithelium (EP) where cell nucleus are visible by DAPI in blue (**b**), nor in the connective tissue (c) (**c**). Pg clusters are present in pathological gingiva (**d**) both in the epithelium (**e**) and in the connective tissue (**f**). These bacteria are found in AAA, particularly in the rupture area (RA) between media (M) and atheroma (Ath) (**h**). The elastic fibers are highlighted in green. By RT-PCR, Pg is detected in dental plaque from periodontitis, whereas they are not in dental plaque from clinically healthy gingiva (G1 and G2) (**g**)

The origin of Pg is the oral cavity. We assessed its presence in Gingiva and dental plaque. Dental plaque retrieved from individuals affected by periodontitis contains Pg (P). This is not the case for non-periodontitis patients (Fig. 4G).

Clinically healthy gingiva did not contain such pathogenic bacteria (Fig. 4A, B, and C). Bacterial clusters were found in periodontitis (Fig. 4D) at both epithelial (Fig. 4E) and connective tissue layers (Fig. 4F). The same bacteria clusters that we have seen in pathological gingiva were also present in the AAA sample which were previously positive. The bacteria were present at injury areas and more particularly around atheroma plaque (Fig. 4H). We did not detect by immunohistochemistry Pg in TAA whereas it was the case for the AAA. They might not be alive in the TAA, but only in the AAA. Therefore, these bacteria might be a parameter to consider in AAA when the GF are co-cultured with aneurysm samples.

GF increases the level of TIMP-1 and MMP-9/TIMP-1 and modulates active MMP-9 and pro-MMP-9 in the AAA and TAA secretions.

In the presence of GF in the AA culture, TIMP-1 secretion increased. The results were significant for TAA at day 7 (p = 0,0159), but at day 14 the variation of the TIMP-1 level in the presence or in the absence of GF in the culture was not significant. For AAA, the increase of TIMP-1 was significant both at day 7 (p = 0,0385) and at day 14 (p = 0,0076).

The presence of GF in the culture of AA led to a higher level of the inhibitory complex MMP-9/TIMP-1. At day 7 and day 14 of the culture of TAA, the presence of GF tended to increase the complex’s concentration. For AAA, GF led to a significant increase of the complex’s concentration at day 7 of culture (p = 0,0313) and at day 14 of culture (p = 0,0465). This inhibitory complex inhibits MMP-9 activity. The important point of GF use is its inhibition of MMP-9.

**Fig. 5:**
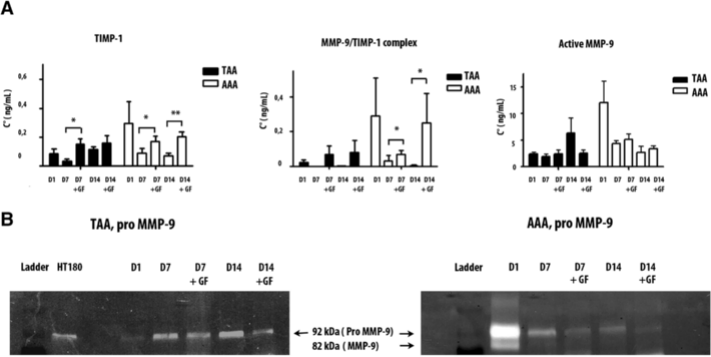
TIMP-1, MMP-9/TIMP-1, active MMP-9 and pro MMP-9 assays. (**a**) GF cocultured with TAA samples lead to an increase of TIMP-1 at day 7 of culture (p = 0.0103), MMP-9/TIMP-1 complexes at day 7 and day 14 of culture (p < 0.0001), and the decrease of active MMP-9 at day 14 of culture. For AAA, there is an increase of TIMP-1 but it is significant only at day 14 (p = 0.004). There is also an increase of MMP-9/TIMP-1 complexes at day 14. There is not significant modification of active MMP-9 for AAA when GF is cocultured with AAA. (**b**) In the zymogram, the presence of GF lead to a decrease of pro MMP-9 for AAA and TAA. * p < 0.1. **p < 0.001. ***p < 0.0001

MMP-9 can be an active or a zymogen form in the medium. We wanted to analyse its presence when GF is co-cultured. The variation of pro MMP-9 was noticed in AAA and TAA samples. Indeed, GF decreased expression level of pro-MMP-9 in TAA and AAA co-cultures at day 7 and day 14 (Fig. 5B). But for AAA, it is not the case for all the samples. 3/5 samples were affected by this decrease of pro-MMP-9. This form is more present at day 1 in AAA culture than in TAA culture. Both lesions seemed different from the beginning. That was confirmed when we tested the level of active MMP-9 in the presence of GF. AAA lesions secreted more active MMP-9 at day 1 than TAA.

When GF was present in the culture, active MMP-9 tended to decrease in TAA cultures at day 14 as opposed to AAA where no such differences were noticed.

The different responses of both lesions to GF and the different level of MMP-9 at day 1 of the culture have conducted us to think of AAA and TAA as two different diseases. Also, the presence of a peridontic bacteria might explain the different responses.

## Discussion

Previous studies using aneurysm animal models have shown an effective decrease in the size of the aortic aneurysms by local injection of GF (Gogly et al. 2007).

However, our animal model of aneurysm in rabbits (Durand et al. 2012) is quite different from the aneurysm pathophysiology in humans. Indeed, the atheroma plaque and potential infectious factors are absent. In addition, two types of aortic aneurysms are described in humans, abdominal aortic aneurysm (AAA) and thoracic aortic aneurysm (TAA). We first wanted to study the histological and biochemical differences between human AAA and TAA. In a second step we have investigated the presence of oral bacteria on our aneurysm samples. Finally, we have re-evaluated the therapeutic capabilities of gingival fibroblasts, neurectodermic human cells, put into co-cultures with AAA and TAA.

Our first results underlined the fact that AAA and TAA are two different entities as proposed in other studies (Guo et al. 2006; Ruddy et al. 2008). Indeed we first have noticed that AAA secreted more MMP-9 than TAA in GF-free cultures. This fact was correlated with the histological staining. We have shown that the tissues were not degraded in the same way. For TAA, the destruction area was localized in media and characterized by the so-called "cystic medial lesion" where we think that acellular separation in small areas developed to the degree that might weaken the aorta wall, resulting in the aneurysm. On the other hand, AAA histology showed larger damaged areas with infiltration of inflammatory cells and almost no remaining elastic fibres. Indeed the inflammatory infiltration has been described earlier in AAA (Takahashi et al. 2001; Ocana et al. 2003; Reeps et al. 2009). In a concomitant way to our study, neovascularisation was observed in AAA atheroma. This has been described by authors as an element linked to the development of aneurysms (Kaneko et al 2011). Furthermore, endothelial cells and SMC of mature neo-vessels have shown to secrete MMPs which may participate to the degradation of the aortic wall (Reeps et al. 2009). The relationship between inflammation and neovascularisation in the progression of AAA has been well-established (Mayranpaa et al. 2009) and was supported by our results. In our study, we have shown that elastic fibers were excessively degraded in AAA with a remarkable formation of fibrosis. Thus, we may propose that the fibrosis observed in the AAA adventitia may compensate for the elastic fiber loss and thus better limits the extent of the pathology which leads finally to more invasive destruction of the media. This is also observed in TAA but to a lesser extent and may be explained by the lower fibrous degradation. Adventitial fibrosis might prevent the enlargement of the wall and delay its rupture. However, as the injury is not treated, the disease is maintained and the local inflammation remains and aggravates proteolysis.

This inflammation was hypothesized here to be aggravated by infectious factors which are found in some AA samples.

We studied this factor because during the AAA cultures, we were often confronted with contamination problems whereas it was never the case for TAA. GF, which is a cell in contact with oral bacteria in the oral cavity, is not affected by these problems when the cells came from clinically healthy gingiva. Moreover, when they were used for co-culture experiment they were pre-treated in a medium containing antibiotic. Otherwise, in a previous in vivo experiment where elastase aneurysms were treated with GF in rabbits, we never noticed infection issues. The fact that there were contaminations in some AAA cultures led us to stop the culture for the samples concerned. As in the literature we found many relations about periodontitis and cardiovascular disease (Yamazaki et al. 2007; Suzuki et al. 2010); we wondered if we could find periodontopathic bacteria in the samples. And indeed, these bacteria were present but Pg was the bacterium found in most aneurysm samples. The presence of this bacterium was only confirmed clearly by immunohistochemistry (protein level) in AAA. Few authors have explored the thoracic aneurysms for periodontal bacteria detection, which can explain why no report was found describing 16sRNA detection and no bacteria were detected by immunohistology. We may hypothesize that this method is not as sensitive as RT-PCR. We only confirmed bacteria presence by immunohistology in AAA, thus agreeing with some reports (Kurihara et al. 2004; Delbosc 2011) and disagreeing with other reports (Marques da Silva et al. 2005). This difference between PCR and protein detection in TAA might arise from bacterial DNA detected from dead or phagocyted bacteria.

Pg, which was the most often detected bacteria in our study, is a very aggressive bacterium which can go through the epithelial-connective barrier and reach the bloodstream. It originates from oral tissues and as other authors have shown, goes into the systemic circulation (Belstrom et al. 2011). Our study shows that it can reach aorta wall. Periodontopathic bacteria are responsible for the periodontium degradation and, by their presence in AAA, they may explain the difference of degradation/severity between AAA and TAA. We think that it may impact the tissue degradation by stimulating cytokines and metalloproteinases. They also may cause by themselves an inflammation (Bodet et al. 2006) or worsen it.

Our last study is to evaluate the effects of GF in cocultures with AAA and TAA and verify the results obtained in animals (Gogly et al. 2009; Gogly et al. 2007). Our work focuses specifically on MMP-9 and TIMP1 whose roles in the aneurismal pathology are particularly studied (Petersen et al. 2000; Barbour et al. 2007; Allaire et al. 1998). Our ex vivo results suggested that the presence of GF in co-culture with aortic aneurysm samples may modify the AA proteolytic processes. For all the studied samples (from abdominal and thoracic lesions), TIMP-1 was increased. This later can complex with MMP-9 and proMMP-9 and inhibits its action of matrix degradation. By this way, MMP-9/TIMP-1 complexes were also increased when GFs were added. However, when we studied the variation of active MMP-9 in the culture, we noticed a tendency of decrease for TAA but not for AAA samples while proMMP-9 seemed to decrease in both AA. Although non-active, zymogens (pro-MMP9) are important since they can be quickly activated and therefore could result in extracellular matrix catabolism. Indeed, most articles describing implication of MMP-9 in pathological processes do not differentiate between the two forms, making it difficult to know which is the most involved in pathologies, although logics make us think that the active form was the most predominant in matrix degradation. However, all MMP-9 isoforms were detected in periodontitis patients’ oral cavity (Makela et al. 1994). In fact, concerning the AAA samples we noticed a remarkable decrease in the zymogram gel for 3 out of 5 samples whereas for TAA all the samples exhibited the decrease of pro MMP-9 when GF was co-cultured.

Finally, for the samples of aneurysms particularly degraded, GF effect might not be efficient. For AAA, MMP-9 was too concentrated at day 1. GF might not produce enough TIMP-1 to inhibit active MMP-9. This fact can be due to the presence of Pg, which might maintain and aggravate the inflammation, or that TIMP-1 has a strong affinity towards proMMP-9, whose quantity decreased in our AAA and TAA experiments. Thus, maybe, the quantity of GF should be increased in AAA compared to TAA. This will need further investigations.

## Conclusion

TAA and AAA are two distinct lesions. AAA wall degradation is very intensive and could be aggravated by the presence of infectious factors. Pg proteins are clearly detected in AAA. Pg is an aggressive bacterium participating in the pathogenesis of periodontitis which strongly activates inflammation and tissue destruction. It could further damage the aneurysm lesion already there, due to the inflammation it induces. Our conclusion is that GF may be an interesting treatment for aortic aneurysm but periodontal care is essential for all patients with abdominal aortic aneurysm or even thoracic aneurysm.

## Additional file

Additional file 1: Table S1. characteristics of the aneurysm samples. The aneurysm are from 4 females (F) and 14 men (M). There are 6 aneurysm samples from thoracic aorta (TAA), and 12 from abdominal aorta (AAA). **Table S2.** Primer sequences. **Table S3.** Detection of oral bacteria in AAA and TAA samples by PCR and Immuno histochemistry (IHC).
