# Supplementary material for: Comparative study of abdominal and thoracic aortic aneurysms: their pathogenesis and a gingival fibroblasts-based ex vivo treatment
Source: Springerplus. 2015 May 16;4:231. doi: 10.1186/s40064-015-0976-9 (PMC4475515; doi:10.1186/s40064-015-0976-9)
Supplement: Additional file 1: Table S1. — characteristics of the aneurysm samples. The aneurysm are from 4 females (F) and 14 men (M). There are 6 aneurysm samples from thoracic aorta (TAA), and 12 from abdominal aorta (AAA). Table S2. Primer sequences. Table S3. Detection of oral bacteria in AAA and TAA samples by PCR and Immuno histochemistry (IHC). [file 40064_2015_976_MOESM1_ESM.doc]

**Tables**

**Table S1 : characteristics of the aneurysm samples**. The aneurysm are from 4 females (F) and 14 men (M). There are 6 aneurysm samples from thoracic aorta (TAA), and 12 from abdominal aorta.

| **Periodontogene** | **Sequences** | **Amplicon Length** |
| --- | --- | --- |
| *Porphyromonas gingivalis 16S* | Forward: ACC TAA CCC GGG ATT GAA ATG  Reverse: CAA CAA TGC AGC ACC TAC ATA GAA | 83pb |
| *Treponema denticola 16S* | Forward: CCG AAT GTG CTC ATT TAC ATA AAG GT  Reverse: GAT ACC CAT CGT TGC CTT GGT | 122pb |
| *Tannerella forsythia 16S* | Forward: ATC CTG GCT CAG GAT GAA CG  Reverse: TAC GCA TAC CCA TCC GCA A | 226pb |
| *Prevotella intermedia 16S* | Forward: TCC ACC GAT GAA TCT TTG GTC  Reverse: ATC CAA CCT TCC CTC CAC TC | 98pb |

| β-actin | Forward: CGC GAG AAG ATG ACC CAG ATC AT  Reverse: ATA GCA CAG CCT GGA TAG CAA CG | 75pb |
| --- | --- | --- |

**Table S2: Primer sequences**

|  | **PCR** | | | | **IHC** |
| --- | --- | --- | --- | --- | --- |
| Samples | Pg | Td | Tf | Pi | Pg |
| AAA1 | + | + | - | - | + |
| AAA2 | + | - | - | - | + |
| AAA3 | + | + | - | - | + |
| AAA4 | - | - | - | - | - |
| AAA5 | + | - | - | - | + |
|  |  |  |  |  |  |
| TAA1 | + | - | - | - | - |
| TAA2 | + | + | - | - | - |
| TAA3 | - | - | - | - | - |
| TAA4 | - | - | - | - | - |
| TAA5 | - | - | - | - | - |
| TAA6 | + | - | - | - | - |
| TAA7 | + | + | + | - | - |

**Table S3: Detection of oral bacteria in AAA and TAA samples by PCR and Immuno histochemistry (IHC).**
